# A Comparative Study of Lesion-Centered and Severity-Based Approaches to Diabetic Retinopathy Classification: Improving Interpretability and Performance

**DOI:** 10.3390/biomedicines13061446

**Published:** 2025-06-12

**Authors:** Gang-Min Park, Ji-Hoon Moon, Ho-Gil Jung

**Affiliations:** 1 Department of Data Science, Seoul National University of Science and Technology, Seoul 01811, Republic of Korea; gangmin.park@seoultech.ac.kr (G.-M.P.); jhmoon34@seoultech.ac.kr (J.-H.M.); 2 Department of Ophthalmology, National Medical Center, Seoul 04564, Republic of Korea

**Keywords:** diabetic retinopathy, fine-grained lesion detection, medical expert labeling, dataset integration, knowledge transfer, lesion-centered labeling, APTOS dataset

## Abstract

**Background:** Despite advances in artificial intelligence (AI) for Diabetic Retinopathy (DR) classification, traditional severity-based approaches often lack interpretability and fail to capture specific lesion-centered characteristics. To address these limitations, we constructed the National Medical Center (NMC) dataset, independently annotated by medical professionals with detailed labels of major DR lesions, including retinal hemorrhages, microaneurysms, and exudates. **Methods:** This study explores four critical research questions. First, we assess the analytical advantages of lesion-centered labeling compared to traditional severity-based labeling. Second, we investigate the potential complementarity between these labeling approaches through integration experiments. Third, we analyze how various model architectures and classification strategies perform under different labeling schemes. Finally, we evaluate decision-making differences between labeling methods using visualization techniques. We benchmarked the lesion-centered NMC dataset against the severity-based public Asia Pacific Tele-Ophthalmology Society (APTOS) dataset, conducting experiments with EfficientNet—a convolutional neural network architecture—and diverse classification strategies. **Results:** Our results demonstrate that binary classification effectively identifies severe non-proliferative Diabetic Retinopathy (Severe NPDR) exhibiting complex lesion patterns, while relationship-based learning enhances performance for underrepresented classes. Transfer learning from NMC to APTOS notably improved severity classification, achieving performance gains of 15.2% in mild cases and 66.3% in severe cases through feature fusion using Bidirectional Feature Pyramid Network (BiFPN) and Feature Pyramid Network (FPN). Visualization results confirmed that lesion-centered models focus more precisely on pathological features. **Conclusions:** Our findings highlight the benefits of integrating lesion-centered and severity-based information to enhance both accuracy and interpretability in DR classification. Future research directions include spatial lesion mapping and the development of clinically grounded learning methodologies.

## 1. Introduction

Diabetic Retinopathy (DR) is a significant microvascular complication of diabetes mellitus, characterized by progressive damage to retinal blood vessels due to prolonged hyperglycemia and affecting approximately 30% of diabetes patients worldwide [1]. As one of the leading causes of preventable blindness, DR’s impact continues to grow with diabetic patients projected to reach 783 million by 2045 [2]. The condition progresses through distinct stages: from mild Non-Proliferative Diabetic Retinopathy (NPDR) with microaneurysms, to severe NPDR with extensive vascular abnormalities, and finally to Proliferative Diabetic Retinopathy (PDR) marked by neovascularization and potential vitreous hemorrhage [3]. These stages manifest various pathological features including microaneurysms, retinal hemorrhages, hard exudates, and cotton wool spots that collectively contribute to vision impairment if left untreated, making early detection and timely intervention crucial for reducing the risk of severe visual impairment [3,4]. The standard approach to DR diagnosis involves clinical examination of fundus images by ophthalmologists, who assess the presence and extent of various retinal lesions to determine disease severity [5]. However, this traditional diagnostic method faces significant challenges. Interpretation and classification of fundus images demand high-level expertise and experience, making the process time-consuming and costly. Moreover, the growing number of ophthalmic patients coupled with the shortage of skilled ophthalmologists creates a substantial gap in healthcare delivery, particularly in underserved regions [5]. These limitations underline the critical need for more efficient and automated analysis tools that can support timely and accurate DR diagnosis.

Artificial Intelligence (AI) has emerged as a promising solution to address these challenges in DR diagnosis [6]. AI-based fundus image analysis allows visual assessment of retinal conditions, supporting early diagnosis and treatment planning for DR [3]. Well-designed AI models can quickly process large volumes of fundus images and provide reliable preliminary assessments, serving as valuable clinical tools that help address ophthalmologist shortages and optimize specialist time allocation for more complex cases requiring expert judgment [4,5,6]. Convolutional Neural Network (CNN)-based AI models for fundus image classification[7] have evolved significantly, effectively learning local features like lesion shapes and colors for DR diagnosis, with notable success in Kaggle’s DR Detection Challenge [8], demonstrating clinical potential [9,10,11,12]. More recently, Vision Transformer (ViT) models [13] have emerged as powerful alternatives, excelling at capturing global contextual information and spatial relationships between pathological features through large-scale pre-trained models, which is particularly advantageous for analyzing complex DR lesion patterns [14,15,16].

Despite advancements in AI models for DR classification, traditional severity-based approaches face significant limitations in clinical application. Current methods primarily focus on overall disease staging without adequately capturing the specific characteristics and distributions of individual lesions that are critical for accurate diagnosis [3,17]. These approaches struggle particularly with distinguishing intermediate stages of DR, where subtle differences in lesion patterns rather than mere presence determine disease progression [18]. Furthermore, severity-based models often lack interpretability, providing limited insights into which specific retinal abnormalities influenced their classification decisions [19]. To address these limitations, a lesion-centered approach offers compelling advantages. By focusing on detailed characterization of specific pathological features—microaneurysms, hemorrhages, exudates, and neovascularization—such approaches can provide more granular and clinically relevant information [20]. Lesion-centered models potentially offer enhanced interpretability by highlighting specific abnormalities that contribute to diagnosis, aligning more closely with ophthalmologists’ diagnostic processes [21]. Additionally, this approach may improve generalization across diverse patient populations and imaging conditions by focusing on fundamental pathological features rather than overall image patterns [22]. The ability to quantify and track specific lesion types and their spatial relationships could also enable more precise monitoring of disease progression and treatment response, ultimately supporting more personalized management strategies for DR patients [14,15].

To address these limitations, we formulated four key research questions aimed at rethinking DR classification through a lesion-centered labeling:

1. What analytical advantages does a detailed lesion-centered independent labeling structure provide for DR classification compared to traditional severity-based labeling approaches?
2. How do model architectures and classification strategies affect each approach?
3. What is the potential for complementarity between lesion-centered and severity-based approaches?
4. What decision-making differences can be identified through visualization techniques?

For empirical examination of these research propositions, we constructed the National Medical Center (NMC) dataset, a proprietary retinal image dataset with independent lesion-centered labeling, and utilized the Asia Pacific Tele-Ophthalmology Society Blindness Detection Challenge (APTOS) dataset, a widely-used public dataset with severity-based labeling. The NMC dataset provides separate annotations for key pathological features such as retinal hemorrhages, microaneurysms, and exudates, enabling more precise analysis than conventional severity-labeled datasets like APTOS. By using both datasets in our study, we can comprehensively investigate the complementary nature of lesion-centered and severity-based approaches to DR classification.

Our work demonstrates that lesion-centered labeling captures critical pathological details often overlooked in severity-based approaches, while integration of both methods enhances overall classification performance. We compare baseline models across different learning approaches to reveal how dataset characteristics influence model performance. Through visualization analysis, we show how lesion-centered models focus on specific pathological indicators rather than general image patterns, providing greater clinical interpretability. These insights advance the development of more precise and explainable AI-based DR diagnostic systems suitable for real-world clinical implementation.

The rest of this paper is organized as follows. In Section 2, we present related research. In Section 3, we analyze and compare the characteristics of APTOS and NMC datasets. In Section 4, we describe motivating experiments for dataset integration. In Section 5, we explain our proposed methodology. In Section 6, we validate the effectiveness of our proposed method through experimental results. In Section 7, we discuss our analysis findings. Finally, in Section 8, we conclude the paper and present future research directions.

## 2. Related Works

### 2.1. Development of DR Diagnostic Models

Deep learning has emerged as a widely adopted methodology in the development of diagnostic models for DR, owing to its capacity to learn complex visual features directly from fundus images.

*CNN-based architectures.* CNNs [7] have historically served as the foundation of early DR classification models. Architectures such as DenseNet121 [23], ResNet [24], and U-Net [25] have demonstrated high performance in fundus image classification, lesion segmentation, and disease staging. For example, Mohanty et al. [26] built a customized DenseNet121-based model that achieved 97.3% classification accuracy on the APTOS 2019 dataset, reflecting the effectiveness of CNNs in DR severity grading. Similarly, Nazir et al. [27] combined DenseNet-100 for feature extraction and CenterNet [28] for lesion localization, yielding enhanced robustness under noisy imaging conditions for both DR and Diabetic Macular Edema (DME). Yıldırım et al. [29] introduced a deep feature engineering framework based on pretrained MobileNetv2 [30] and nested patch division, targeting treatment-oriented DR classification using fundus angiography images. In addition, Kobat et al. [31] proposed a patch-based DR classification framework using horizontally and vertically divided fundus images, with feature extraction via pretrained DenseNet201 and classification using a cubic SVM [32]. In contrast to end-to-end CNN models, feature-level frameworks such as Barua et al. [33] combine deep features from multiple pretrained CNNs and use classical machine learning classifiers for retinal disease detection.

*Transformer-based architectures.* ViT [13] introduced the use of self-attention in computer vision, replacing convolutional operations with token-wise attention. Unlike traditional CNNs, these models leverage self-attention mechanisms to emphasize crucial image regions, demonstrating superior performance in medical image analysis [34,35,36]. In ophthalmology, ViT variants such as Swin-ViT [37] and MaxViT [38] have shown superior performance by capturing hierarchical and multi-scale features. Swin-ViT retains global context while incorporating local inductive biases, making it suitable for low-resolution fundus images. MaxViT further improves performance on high-resolution medical images by integrating both grid-based and axial attention into a unified framework. These models demonstrate high performance even with low-resolution or limited data settings and offer training methodologies optimized for data-scarce environments [39,40].

Given the structural differences between CNNs and Transformers, we hypothesize that each model may exhibit different sensitivities depending on the label structure of the dataset. Specifically, CNNs such as EfficientNet [41] may be more effective at detecting localized features aligned with lesion-centered labels, while Swin Transformer’s hierarchical attention mechanisms may align better with global, severity-based annotations.

### 2.2. Dataset Structure and Labeling Strategy

The structure of dataset labels in DR diagnosis significantly influences model learning behavior. In this domain, two labeling approaches are commonly adopted: severity-based [42,43] and lesion-centered labeling [44].

*Severity-based labeling:* severity-based datasets such as APTOS 2019 [43] provide a discrete ordinal scale for disease severity, ranging from 0 (no DR) to 4 (proliferative DR). These labels align closely with clinical diagnostic guidelines and are often used in classification tasks that focus on disease staging. However, this approach may obscure fine-grained lesion-centered information and is affected by inter-grader variability. Additionally, severity-based datasets frequently suffer from class imbalance, which can hinder model training stability and generalization [45].

*Lesion-centered labeling:* In contrast, lesion-centered datasets, such as the Indian Diabetic Retinopathy Image Dataset (IDRiD) [44], annotate the presence and location of specific pathological findings, including microaneurysms, hemorrhages, soft and hard exudates. This multi-label format more closely reflects real-world clinical settings, where multiple lesions may coexist in a single fundus image. While lesion-centered labeling enables more granular supervision and facilitates explainable predictions, it is often annotation-intensive due to the need for pixel-level lesion segmentation. Moreover, label noise may arise from inter-observer variability in delineating lesion boundaries. The co-occurrence of rare lesions and the complexity of fine-grained annotation may also introduce class imbalance and inconsistency, particularly when labels are derived from expert interpretation across diverse imaging conditions. Lesion-centered datasets often enable better model interpretability through visualization techniques like Gradient-weighted Class Activation Mapping (GradCAM) [46] and Integrated Gradients (IG) [47]. These methods show that models trained on detailed lesion labels tend to focus on clinically meaningful regions, although such attention does not always guarantee better classification performance.

### 2.3. Multi-Label Classification Strategies

Multi-label classification has gained increasing attention in DR diagnosis, as multiple pathological findings can coexist within a single fundus image. Unlike single-label classification, which assigns one global severity level per image, multi-label classification enables models to detect and quantify multiple lesions simultaneously, allowing for more fine-grained analysis and better alignment with real-world clinical practice.

*Architectural advances.* Wang et al. [48] demonstrated that vision transformer models are effective in predicting multiple ocular diseases simultaneously, confirming their applicability in multi-label eye disease classification. These studies have also explored architectural strategies to improve label-wise prediction, particularly through multi-head classifiers. Jain et al. [49] employed a multi-head classification approach that independently predicts each label using separate output branches, showing improved performance over single-head models by mitigating label interaction effects. Du et al. [50] introduced Multi-Path Vision Transformer (MPViT), which enhances multi-disease classification by extracting independent label-specific representations through multiple attention paths and parallel patch embeddings. Additionally, classifier chain models have also been proposed to capture label dependencies by predicting labels sequentially. This approach has shown improved performance in settings with interdependent lesions or class imbalance [51].

*Optimization strategies.* Beyond architectural innovations, learning strategies have also been developed to address the unique challenges of multi-label classification, such as class imbalance and inter-label redundancy. These challenges are especially prevalent in medical imaging domains, including but not limited to DR datasets. Ridnik et al. [52] proposed an asymmetric loss function that down-weights the influence of negative classes during training, thereby mitigating over-suppression and enhancing recall for rare positive labels—a frequent issue in lesion-centered datasets. Complementarily, Hang and Zhang [53] introduced a dual-perspective feature learning framework that explicitly models label-specific features from both inclusion and exclusion perspectives. By learning what defines a label as well as what distinguishes it from others, this approach enhances inter-label discrimination and reduces prediction overlap, which is especially beneficial in medical images where lesions frequently co-occur.

### 2.4. Knowledge Transfer and Dataset Integration Strategies

In medical imaging tasks such as DR diagnosis, dataset heterogeneity often arises due to differences in labeling criteria, image acquisition settings, and population demographics. These inconsistencies can degrade the generalizability of models trained on a single dataset. To address this, various strategies have been proposed for cross-domain knowledge transfer and dataset integration.

*Domain adaptation strategies.* Zhang et al. [54] proposed Collaborative Unsupervised Domain Adaptation (CoUDA), a framework designed to address distribution mismatch between public and local datasets in medical imaging. The method trains two peer networks on source and target domains with noisy pseudo-label exchanges and enforces consistency via shared co-adaptation layers. Liang et al. [55] addressed test-time distribution shifts through a self-supervised test-time adaptation strategy. Their method leverages entropy minimization and consistency regularization using augmented views of the input image, allowing the model to adapt to target distributions during inference. Complementarily, Valanarasu et al. [56] proposed FLY-TTA, a lightweight test-time adaptation framework that adapts model behavior using adaptive batch normalization and domain-specific priors without requiring access to source data or gradient updates.

*Augmentation and representation learning.* To promote robustness across heterogeneous datasets, Kebaili et al. [57] explored generative augmentation techniques. Their work evaluated autoencoder-based reconstruction, GAN-based synthesis, and diffusion model-based transformation to simulate diverse imaging styles and improve representation invariance.

*Semi-supervised learning under label scarcity.* In addition to domain adaptation, semi-supervised learning techniques have also been investigated. Huynh et al. [58] proposed Adaptive Blend Consistency Loss (ABCL), a novel regularization method that adaptively blends class distributions from original and augmented unlabeled samples. ABCL improves learning stability under class imbalance and noisy labels, especially in data-scarce medical environments.

*Imbalance-aware training.* Lastly, Bria et al. [45] tackled extreme class imbalance by introducing a two-stage cascaded learning framework. Background-dominant samples are filtered using a decision tree cascade and rebalanced before being passed to a CNN classifier, increasing sensitivity to clinically important yet underrepresented lesions.

While these methods offer robust solutions to dataset heterogeneity, most focus on either image distribution or annotation quality. Few studies have explicitly examined how differences in labeling strategy—such as severity-based versus lesion-centered structures—interact with model architecture during knowledge transfer. The study addresses this gap through cross-dataset evaluation using both CNNs and Transformer models trained on structurally distinct DR datasets.

## 3. DataSets

This study utilizes two primary datasets for DR classification: the APTOS and the NMC datasets. Each dataset provides diverse characteristics and labeling criteria required for DR classification and is used complementarily based on the research objectives. This section provides a detailed explanation of the composition and features of these two datasets.

Vascular changes within the retina cause DR and can be classified into two main types: NPDR and PDR. NPDR represents the early stages and is further subdivided into mild, moderate, and severe categories based on the extent of lesion progression. Severe NPDR is particularly identified using the “4-2-1 rule”, where any one of these criteria indicates high risk: hemorrhages in all four quadrants, venous beading in two or more quadrants, or Intraretinal Microvascular Abnormalities (IRMA) in at least one quadrant. IRMA represents abnormal branching or dilation of existing retinal blood vessels and serves as an important marker for disease progression. PDR involves more severe lesions characterized by features such as neovascularization (the growth of new, fragile blood vessels) and vitreous/preretinal hemorrhage. Table 1 summarizes the key features of each stage of DR. This staged classification of DR is directly reflected in the labeling approaches of the APTOS and NMC datasets, serving as a crucial foundation for understanding the composition and differences between the datasets.

### 3.1. APTOS

**Figure 1:**
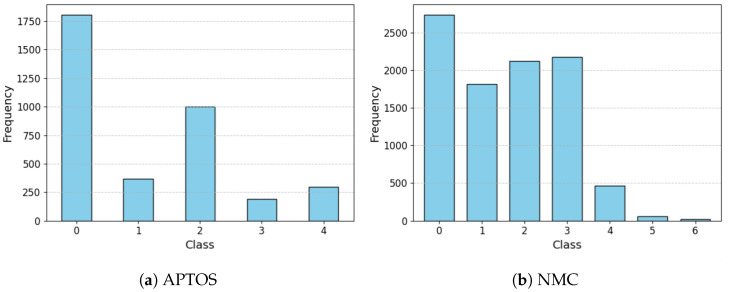
Distribution of Fundus Image Datasets.

**Figure 2:**
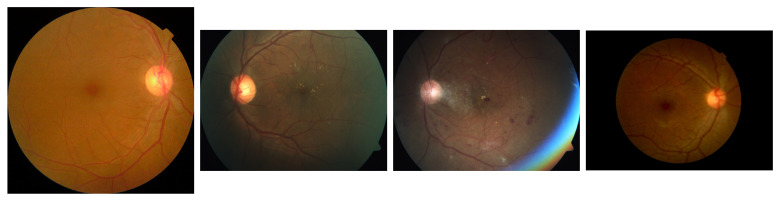
Representative fundus images from the APTOS dataset, demonstrating variations in image quality, illumination, and pathological severity.

The APTOS [43] dataset represents a significant public resource for DR classification, made available through Kaggle competitions. Comprising approximately 3662 fundus images with diverse resolutions and quality levels, this dataset classifies DR severity into five stages (0∼4) based on the Early Treatment Diabetic Retinopathy Study (ETDRS) [59] criteria and is widely adopted in DR classification research. The APTOS labeling system follows a straightforward structure: No DR (0), Mild DR (1), Moderate DR (2), Severe DR (3), and Proliferative DR (4). The class distribution within the APTOS dataset is illustrated in Figure 1a. Examples of fundus images from the APTOS are illustrated in Figure 2.

While the APTOS dataset’s strength lies in its inclusion of varying image qualities and diverse patient populations, it presents certain limitations in fully representing natural clinical environments. The single-label structure constrains the learning and analysis of complex interactions between DR lesions, and the absence of detailed lesion information within severity labels has been noted as a significant limitation. Despite these constraints, the APTOS dataset proves valuable for initial model development and training in DR classification, mainly due to its standardized labeling criteria that facilitate model performance comparisons. However, its limitations in capturing clinical complexity suggest the need for supplementary datasets to provide more comprehensive coverage of DR manifestations.

### 3.2. NMC

**Figure 3:**
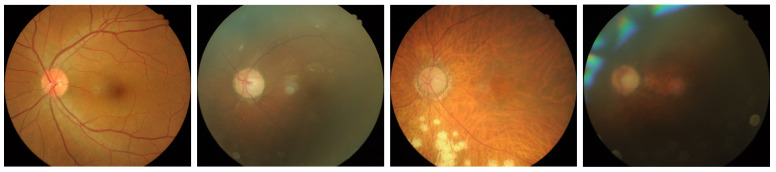
Representative fundus images from the NMC dataset, showcasing diverse lesion types and varying imaging conditions across different stages of Diabetic Retinopathy.

The NMC dataset is a local dataset for DR classification distinguished by its multi-label structure incorporating detailed lesion information. Unlike APTOS, this dataset captures the direct cause of DR through comprehensive lesion-centered labeling by experienced ophthalmologists. The data was collected from subjects of various ages and both genders, ensuring demographic diversity. Images were captured using a KOWA Nonmyd-7 fundus camera fixed at positions optimized for examination, and was used for both mydriatic and non-mydriatic examinations. The images were taken by three different examiners in rotation, while the labeling of all fundus photographs was exclusively conducted by a single designated ophthalmologist to ensure consistency. The NMC dataset was collected and annotated under the approval of the Institutional Review Board of National Medical Center (Approval Code: NMC-2024-03-034, 28 March 2024) in accordance with the Declaration of Helsinki. Patient informed consent was waived due to the retrospective nature of the study using fully anonymized retinal images. Comprising approximately 6500 high-resolution fundus images with varying quality, the NMC dataset allows multiple labels per image. The labeling system categorizes various DR lesion conditions, as detailed in Table 2. After excluding labels 8, 9, and 10 (which do not impact DR classification) and removing noisy labels (where normal and DR conditions coexist), the final dataset contains approximately 6000 samples with 27 unique label combinations. Figure 1b presents the frequency distribution of these class combinations. The NMC dataset is anonymized to ensure that all patient identification information is removed, complying with ethical standards for medical data usage. Examples of fundus images from the APTOS are illustrated in Figure 3.

The multi-label structure of the NMC dataset enables single images to carry multiple lesion labels, facilitating effective modeling of complex DR lesion patterns and interactions observed in clinical settings. This detailed lesion information provides opportunities for more comprehensive analysis compared to the simplified severity labels in APTOS, making these datasets complementary. However, the NMC dataset has certain limitations: it does not specify lesion quantities, locations, or sizes. Furthermore, some lesion labels not directly associated with DR progression (such as laser scars and media opacity) may require exclusion during model training.

Table 3 summarizes a comparison of the APTOS and NMC datasets. Both datasets have unique strengths and limitations and are used complementarily depending on the research objectives. The APTOS dataset provides simplified severity labels, making it useful for model performance comparison, but it fails to capture sufficient clinical details. In contrast, the NMC dataset is well-suited for analysis and model development based on real clinical data, thanks to its detailed lesion labeling. In this study, we leverage both datasets to adopt a multifaceted approach to DR classification and explore the potential for dataset integration. For brevity and consistency throughout the results section, we introduce label abbreviations for the major lesion types in the NMC dataset. The abbreviations are as follows: HE—Retinal hemorrhages, MA—Microaneurysms, EX—Exudates, CWS–Cotton wool spots, VH—Vitreous hemorrhages, PRH—Preretinal hemorrhages, and NA—No abnormality (normal cases). These abbreviations are used throughout the experimental sections to ensure concise and consistent representation of the NMC dataset. A full description of each class is provided in Table 2.

## 4. Motivating Experiments

To explore the feasibility of integrating lesion-centered and severity-based DR classification approaches, we conducted preliminary transfer learning experiments. These experiments were designed to assess the complementary nature of two structurally distinct datasets—APTOS [43] and NMC—which differ in labeling strategy, class distribution, and annotation granularity. We first established baseline models by training each dataset independently, then used these baseline models for cross-dataset fine-tuning to evaluate directional transferability.

Our motivating experimental results showed asymmetric and class-specific performance shifts across the two datasets. Transferring lesion-centered knowledge from NMC to the severity-based APTOS task resulted in a modest decrease in overall Micro F1-score, yet certain DR stages benefitted from lesion-centered representations. In particular, Severe DR improved substantially by 0.098, and Mild DR by 0.071. Conversely, transferring severity-based knowledge from APTOS to the lesion-centered NMC task led to a slight increase in the overall Micro F1-score, with certain lesion types benefitting from severity-based priors. Notably, Vitreous Hemorrhage improved by 0.048, and Microaneurysms by 0.012. However, performance declined significantly for Cotton Wool Spots, dropping by 0.075. Detailed experimental results can be found in Section 6.

These findings reinforce that while the lesion-centered and severity-based paradigms provide complementary information, direct transfer learning between them is insufficient to fully bridge their representational gap. In the following section, we present our proposed methodology, which introduces an integrated framework designed to overcome these challenges and to maximize the clinical relevance and generalizability of DR classification models.

## 5. Methodology

To verify the performance of the lesion-centered NMC dataset and enhance its complementarity with the severity-based APTOS [43] dataset, we propose four experimental strategies directly addressing our research questions (RQ). First, for examining the analytical advantages of lesion-centered labeling (RQ1), we focus on model selection to identify suitable models for capturing detailed pathological features. Second, to understand how model architectures and classification strategies affect each approach (RQ2), we explore different classification strategies tailored to single-label and multi-label structures. Third, to investigate the complementary potential between approaches (RQ3), we develop cross-dataset knowledge transfer strategies. Finally, to identify decision-making differences (RQ4), we employ Visualization for Model Focusing techniques to confirm whether models attend to clinically relevant features.

### 5.1. Model Selection

Unlike natural images, fundus images require the extraction of fine-grained features for severity classification and lesion detection. To address our first research question regarding the analytical advantages of lesion-centered labeling, we selected established general-purpose models rather than architectures optimized explicitly for a particular dataset. Two models were chosen for their strengths in detailed feature extraction.

EfficientNet_v2 [60] is a CNN-based model designed to achieve both lightweight architecture and high performance, leveraging MBConv and Fused-MBConv structures [30,61] to maximize computational efficiency and representative capacity. This model effectively learns fine-grained features of the image (e.g., lesion boundaries and color variations) through a hierarchical feature map structure. In addition, it can efficiently adjust its size by applying compound scaling, which balances the network depth and width. EfficientNet_v2 is evaluated as highly suitable for learning subtle features, such as lesion size and shape changes, which are critical for severity classification. Specifically, we employed the EfficientNet_v2_m variant for our experiments. This contains approximately 54.1 M parameters with over 60 convolutional layers utilizing a combination of MBConv and Fused-MBConv blocks. It is optimized for 480 × 480 pixel inputs and requires about 24.7B floating-point operations (FLOPs) for inference.

Swin Transformer [37] is a transformer-based model developed to overcome the limitations of ViT, enabling simultaneous learning of global and local image patterns. Using the Shifted Window Mechanism facilitates interactions between consecutive windows, which is advantageous for learning relative positions and sizes of lesions in DR datasets. Its hierarchical feature map structure enables effective learning of features across various scales, offering better computational efficiency and training stability than traditional ViT models. Swin Transformer demonstrates strengths in capturing global lesion patterns (e.g., distances and interactions between lesions) and their correlations with severity classification. For our implementation, we utilized the Swin_S variant, which comprises approximately 49.6 M parameters organized into 4 stages with a total of 24 transformer blocks. This model processes 224 × 224 pixel inputs using a 7 × 7 window size and 96 embedding dimensions, requiring about 8.7 G FLOPs for inference. Both models were selected based on their comparable parameter counts (approximately 50 M parameters each), allowing for a fair architectural comparison between CNNs and transformer-based approaches while maintaining reasonable computational requirements for medical image analysis.

We employed the micro F1-score as our primary metric for objective performance evaluation. The micro F1-score was chosen because it provides a balanced assessment of model performance in both single-label and multi-label classification scenarios. It is particularly suitable for medical image analysis where class imbalance is common. This metric aggregates the contributions of each class instance to compute the overall F1 score, making it especially valuable for our dataset, where certain severity levels or lesion types may be underrepresented. The micro F1-score is calculated as the harmonic mean of precision and recall, where true positives, false positives, and false negatives are computed globally across all classes rather than for each class independently. This approach ensures that each instance contributes equally to the final score, regardless of its class membership, providing a robust evaluation of the model’s overall classification performance.

### 5.2. Classification Strategy

We note that the APTOS dataset and the NMC dataset differ in their fundamental classification structure. To address our second research question on how model architectures and classification strategies affect each approach, we explore different classification methods appropriate for each dataset structure. The APTOS dataset employs a single-label classification approach, where each fundus image is assigned precisely one severity grade (0∼4). This classification structure is based on mutually exclusive labeling across severity levels—even when multiple lesions are present in an image, they are holistically evaluated to determine a single, comprehensive severity grade. This approach guides the model to learn clear distinctions between severity levels through a unified classifier, as each image’s features contribute to precisely one class prediction.

**Figure 4:**
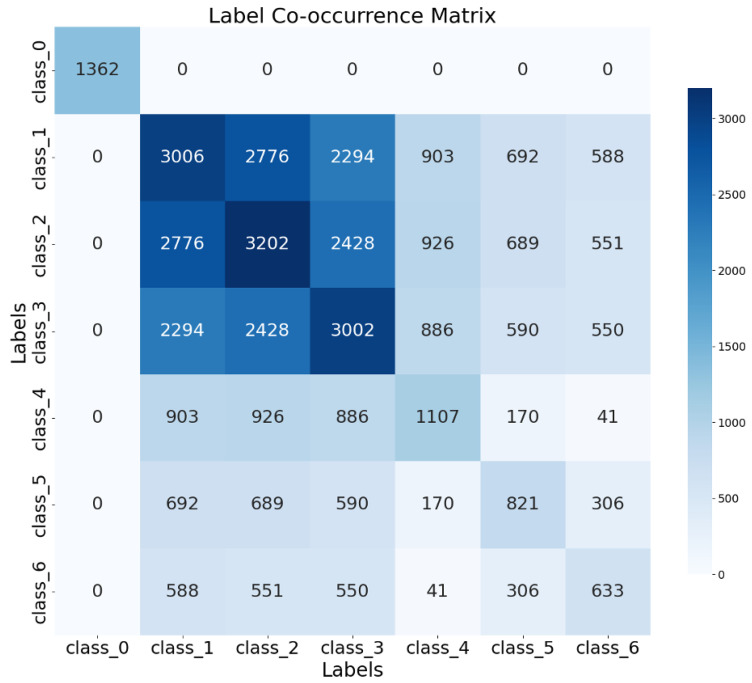
Class confusion matrix of NMC dataset.

In contrast, the NMC dataset is structured as a multi-label classification problem, allowing multiple specific lesion types to be simultaneously present in a single image. While the dataset captures the co-occurrence of different lesions, the traditional multi-label classification approach treats each lesion type as a separate classification task with independent sigmoid output functions. This modeling approach enables the model to learn specific features for each lesion type but does not explicitly incorporate the potential clinical relationships between different lesions. Figure 4 illustrates the correlations between labels in the NMC dataset, revealing that certain lesion types frequently co-occur, suggesting underlying pathophysiological relationships.

These structural differences between datasets necessitate different classification strategies. For the APTOS dataset, we compare two approaches: multi-class classification (where all severity grades are predicted simultaneously) and binary classification for individual classes. This comparison allows us to analyze the impact of interrelationships among severity labels on model performance. While multi-class classification captures the ordinal relationships between severity levels, binary classification allows the model to focus independently on the features of each severity level.

For the NMC dataset, we compare traditional multi-label classification with the label-chain technique, which considers dependencies between labels. Label-chain is a method that sequentially models label dependencies by incorporating the prediction of the previous label into the prediction of the next [51]. This approach enables the learning process to reflect potential clinical relationships between DR lesions, such as the pathological connections between retinal hemorrhages (Class 1) and microaneurysms (Class 2).

### 5.3. Cross-Dataset Knowledge Transfer

We propose two experimental strategies to leverage the complementary characteristics of the APTOS dataset and the NMC, directly addressing our third research question on the potential complementarity between lesion-centered and severity-based approaches. The first strategy aims to improve severity classification performance in the APTOS dataset by fusing features extracted from models trained independently on each dataset. To achieve this, we train the fused features using networks such as the Feature Pyramid Network (FPN) [62] or the Bi-directional Feature Pyramid Network (BiFPN) [63].

FPN is designed to effectively integrate features of different scales by combining the semantic features of higher layers with the detailed features of lower layers through a top-down pathway. This structure excels in capturing objects or features of varying sizes, making it particularly suitable for medical image analysis tasks like DR diagnosis, where lesions of diverse sizes are present. BiFPN extends FPN by introducing bidirectional feature propagation and weighted feature fusion, which enhances performance. It expands the unidirectional information flow in FPN to bidirectional and adjusts the importance of each feature map with learnable weights, enabling more effective feature integration. This approach efficiently utilizes multi-scale lesion information critical for DR diagnosis. This approach uses the detailed lesion information in the NMC dataset to provide additional representational power necessary for severity classification in the APTOS dataset. For instance, the lesion-centered features extracted from the NMC dataset can contribute to learning the features required for severity labels in the APTOS dataset. We explored both unidirectional (FPN) and bidirectional (BiFPN) fusion approaches to ensure comprehensive feature integration across the diverse characteristics of DR classes.

The second strategy involves selecting mapped classes between the APTOS dataset and the NMC dataset to train models on independent binary classification tasks for each class, followed by fine-tuning on each other’s datasets. Table 4 presents a mapping between APTOS severity classes and NMC lesion classes that we defined for our experimental purposes. The individual class definitions themselves follow established clinical guidelines [59], but the specific mapping relationships between these two classification schemes were created based on the clinical characteristics of each class. For example, APTOS Class 2 (Moderate DR) can correspond to NMC Class 1 (Retinal Hemorrhages) when four or more hemorrhages are present in a single hemisphere. Also, APTOS Class 3 (Severe DR) can map to a scenario where NMC Classes 1 (Retinal Hemorrhages) and 2 (Microaneurysms) are present across all quadrants. Additionally, NMC Class 3 (Exudates) and Class 4 (Cotton Wool Spots) are frequently observed as signs of NPDR [64,65,66], but they are not defined as independent labels within APTOS severity classes. However, they remain clinically relevant to DR and may serve as auxiliary features indirectly influencing severity classification.

Meanwhile, the lesion labeling in the NMC dataset does not include information about lesion counts or locations (e.g., specific hemispheres or quadrants), which makes precise mapping to APTOS severity labels challenging. For instance, APTOS Class 2 and Class 3 differ based on the distribution and location of lesions, but the NMC dataset does not capture such spatial details. This limitation restricts the accurate reflection of the nuanced criteria used in the APTOS severity classification. Additional conditions or preprocessing strategies are necessary to address these challenges and bridge the gap between the datasets.

This experiment aims to evaluate whether NMC’s lesion-centered labeling can contribute to learning severity labels in the public dataset and to improve performance by integratively learning the relationships between the two datasets through fine-tuning. Although the NMC dataset does not capture information about lesion size or location, we aim to assess whether models can learn specific detailed lesion characteristics through training, and whether this knowledge can help in discriminating severity levels that are determined by the same lesions. Through this, we confirm the possibility that detailed lesion information of NMC will not remain in a simple auxiliary role but will be utilized as a new feature in the severity classification of APTOS.

### 5.4. Visualization for Model Focusing

In this study, we apply visualization techniques using GradCAM [46] and IG [47] to analyze the learning patterns of our models, addressing our fourth research question regarding decision-making differences between approaches. These two methods are widely used in medical image AI analysis for their ability to provide complementary insights into model decision-making processes. GradCAM highlights broader regions of interest by utilizing the final convolutional layer, providing clinicians with an intuitive understanding of which anatomical areas contribute to the model’s prediction. Meanwhile, IG offers pixel-level precision by evaluating the influence of each pixel across all network layers, which is crucial for detecting subtle DR lesions such as microaneurysms or small hemorrhages. This dual approach is particularly valuable in clinical contexts, where both the overall affected regions and specific pathological details must be accurately identified.

By employing both techniques, we can validate whether the model focuses on clinically relevant features and provide more comprehensive evidence for clinical interpretation. This visualization approach not only evaluates whether the learned features align with the intended objectives but also serves as a vital tool for analyzing the impact of cross-dataset knowledge transfer on the model’s learning outcomes, enabling a visual assessment of the effectiveness and limitations of our complementary learning strategies between datasets.

## 6. Experiments

### 6.1. Experimental Setting

Our experiments were designed to validate the four methodological strategies proposed in Section 5: Model Selection, Classification Strategy, Cross-dataset Knowledge Transfer, and Visualization for Model Focusing. All experiments were conducted using the PyTorch 2.4.1 (with CUDA 12.1 support), with training performed on an NVIDIA A6000 GPU. Input images were resized to 256 × 256, and data augmentation techniques included horizontal flipping, random rotations within 10 degrees, and color jitter adjustments for brightness, contrast, and saturation within a range of 0.2. All images were normalized using ImageNet [67] mean and standard deviation. For both EfficientNet_v2_m and Swin_s models, we utilized the pre-trained weights from the official PyTorch torchvision library (torchvision.models.efficientnet_v2_m and swin_s). The models were trained with a batch size of 32 and an initial learning rate of $1\;\times \;10^{-4}$. The AdamW optimizer was employed with a weight decay of $1\;\times \;10^{-4}$ to prevent overfitting. To stabilize training and promote convergence, a ReduceLROnPlateau scheduler was used to reduce the learning rate by a factor of 0.1 when the validation F1 score plateaued for five consecutive epochs. In addition, early stopping was triggered if no performance improvement was observed for seven epochs.

A key challenge in medical imaging is class imbalance, especially the underrepresentation of advanced disease stages. To address this, a balanced batch sampling strategy was adopted to ensure that each mini-batch contained a proportionate distribution of all classes, enabling the model to learn rare classes more effectively. We chose balanced batch sampling because it naturally handles complex co-occurrence patterns in multi-label scenarios, maintains methodological consistency across transfer learning experiments, and reduces the need for extensive hyperparameter tuning, thereby avoiding additional variables that could confound the transfer learning effects we aimed to evaluate, rather than alternative approaches (e.g., focal loss, oversampling). An epoch was defined as one complete pass over the dataset, with training/validation/test splits set to 70%, 15%, and 15%, respectively, for both the APTOS [43] and NMC datasets. All results presented in this study were obtained exclusively from these independent test sets, which were completely isolated from model training and hyperparameter tuning processes. This consistent experimental protocol serves as the foundation for comparative evaluations across all classification strategies and model architectures. Importantly, the use of strong regularization and balancing techniques ensures that observed differences in performance are attributable to model design and data characteristics rather than confounding factors in the training process. From a clinical perspective, these settings help ensure that high-risk but underrepresented cases—such as severe proliferative DR or rare lesion types—are not systematically overlooked during model training. To simplify the presentation of lesion-centered results, we adopt the NMC label abbreviations (e.g., HE, MA, EX) as introduced in the Section 3.

### 6.2. Motivating Experiments

We established baseline models by training each dataset independently and then used these for cross-dataset fine-tuning to evaluate transfer learning between lesion-centered and severity-based classification approaches. The APTOS dataset was framed as a single-label classification task with softmax output over five DR stages, whereas the NMC dataset was treated as a multi-label classification problem using sigmoid outputs across seven lesion types.

As summarized in Table 5 and Table 6, the bidirectional transfer learning experiments revealed asymmetric and class-specific performance shifts. Transferring knowledge from NMC to APTOS (Table 5) resulted in overall F1-score decrease from 0.8339 to 0.8248, representing a decline of 0.009. However, significant improvements were observed in Severe DR with F1-score improvement of 0.098 and Mild DR with F1-score improvement of 0.071. Conversely, transferring from APTOS to NMC (Table 6) led to a slight increase in overall F1-score from 0.8321 to 0.8338, an improvement of 0.002, with improvements in Microaneurysms of 0.012 and Vitreous Hemorrhage of 0.048, but substantial decline in Cotton Wool Spots of 0.075. These results confirm that while there are complementary benefits between the two classification paradigms, simple transfer learning alone cannot fully bridge their representational gap, necessitating the more sophisticated integrated approach presented in this paper.

### 6.3. Model Selection

The comparative evaluation of model backbones was designed to identify an architecture that balances accuracy and clinical relevance across datasets with distinct label structures. We benchmarked two representative models—EfficientNet_v2_m and Swin Transformer (Swin_s)—under classification paradigms matched to dataset characteristics: single-label classification for APTOS and multi-label classification for NMC. Both models were initialized with ImageNet pre-trained weights [67] and trained under identical settings to ensure a fair comparison.

Table 7 and Table 8 present the micro F1-scores per class for both APTOS and NMC datasets. On the APTOS dataset (Table 7), EfficientNet demonstrated superior performance in three out of five classes, including a notable advantage of 0.138 in Class 4 (Proliferative DR), suggesting its effectiveness in identifying advanced disease stages. In contrast, Swin Transformer slightly outperformed EfficientNet in Class 3 (Severe NPDR) and Class 1 (Mild NPDR), with respective gains of 0.030 and 0.029. The overall average F1-score for EfficientNet was 0.8339, representing an improvement of 0.023 over Swin Transformer. For the NMC dataset (Table 8), where multiple lesions may co-occur, the overall performance difference between the two models was minimal at 0.002. However, in Class 5 (Vitreous Hemorrhage), EfficientNet achieved a micro F1-score of 0.6667, compared to 0.5714 for Swin Transformer—an advantage of 0.095. This result may reflect EfficientNet’s relative robustness in detecting underrepresented yet clinically significant lesion types, although the small sample size for this class limits definitive conclusions.

From a clinical perspective, EfficientNet’s advantage in recognizing late-stage DR (Class 4 in APTOS and Class 5 in NMC) is especially important. These classes correspond to high-risk conditions where timely intervention can prevent vision loss. The model’s superior sensitivity to these categories justifies its selection for subsequent experiments, particularly in applications where early detection of severe pathology is critical.

### 6.4. Classification Strategy

To explore how different classification strategies interact with disease severity representation and lesion co-occurrence, we applied tailored approaches to the APTOS and NMC datasets. Specifically, we compared multi-class and binary classification strategies on the APTOS dataset, where each image is labeled with a single DR severity grade. In contrast, for the NMC dataset, which assigns multiple lesion labels per image, we contrasted standard multi-label classification with a label-chain approach.

As shown in Table 9 and Table 10, the APTOS dataset (Table 9) demonstrated class-specific differences in classification performance when comparing multi-class and binary classification strategies. For Class 3, which exhibits complex lesion patterns, binary classification outperformed multi-class classification by 0.067, likely because the model could independently learn the co-occurrence of multiple lesions described by the 4-2-1 rule (severe intraretinal hemorrhages, venous beading, and IRMA). In contrast, Class 4 (PDR), characterized by distinct pathological signs such as neovascularization and vitreous or preretinal hemorrhage, showed a 0.053 improvement under the multi-class approach. For simpler classes, such as Class 0 (No DR) and Class 2 (Moderate NPDR), the performance difference between the two strategies was minimal. These results suggest that lesion complexity and data imbalance are critical factors in selecting an appropriate classification strategy. Likewise, the NMC dataset (Table 10) exhibited mixed outcomes when comparing conventional multi-label classification and the label-chain approach, with class-specific variations in performance gains and losses. For Class 1 (Retinal Hemorrhages), label-chain learning yielded a performance gain of 0.027. Similarly, for Class 5 (Vitreous Hemorrhages), the improvement was the most pronounced, with an F1-score increase of 0.048. In contrast, Classes 2 (Microaneurysms) and 3 (Hard Exudates) showed slight performance declines under the label-chain strategy of 0.009 and 0.029, respectively. These results highlight that while label-chain learning offers modest improvements for minority classes (Classes 4, 5, 6), rare lesion detection remains a fundamental challenge in lesion-centered classification approaches.

These results suggest that the effectiveness of classification strategies varies depending on the frequency and structural characteristics of each lesion class. Specifically, label-chain learning—an instance of relation-based classification that explicitly models inter-label dependencies—appears beneficial in cases where class imbalance is severe and co-occurrence patterns are informative. This finding highlights the importance of aligning the classification strategy with the data distribution and inter-label structure when designing DR diagnostic models.

### 6.5. Cross-Dataset Knowledge Transfer

The APTOS and NMC datasets can provide complementary perspectives on Diabetic Retinopathy: APTOS captures holistic severity grading, whereas NMC offers detailed lesion-level annotations. However, their structural differences—such as label format, annotation granularity, and classification objectives—make unified modeling nontrivial. In previous experiments, we observed that the effectiveness of classification strategies (e.g., multi-class vs. binary classification, and multi-label vs. label-chain learning) varied across datasets and classes, depending on lesion complexity and class imbalance. In particular, the study on labeling strategies demonstrated that independent feature learning was effective for certain classes, though further improvements are needed for heavily imbalanced categories. To address this, we investigate whether cross-dataset knowledge transfer can bridge these gaps and improve overall model generalization. Specifically, we explore two approaches: (1) feature-level fusion using pre-trained encoders with FPN or BiFPN, and (2) label-aligned fine-tuning based on class mappings across the two datasets.

In the first approach, models pre-trained independently on the APTOS and NMC datasets were used as feature extractors. The extracted representations were then concatenated and passed through integration networks—specifically, FPN and BiFPN—to assess the impact of multi-scale fusion. In the second approach, models pre-trained on one dataset were fine-tuned on the other, based on manually aligned class mappings between severity levels and lesion types, to enable cross-domain adaptation.

The results of the feature fusion experiment are presented in Table 11. Although the overall performance slightly declined compared to the baseline APTOS model (0.8339 → 0.8136 for FPN, 0.8139 for BiFPN), notable improvements were observed in specific classes. BiFPN improved Class 1 (Mild NPDR) by 0.078, and FPN improved Class 3 (Severe NPDR) by 0.185. These improvements closely reflect the label mapping between the datasets: Class 1 in APTOS corresponds to microaneurysms (Class 2 in NMC), and Class 3 corresponds to a combination of hemorrhages and microaneurysms (NMC Classes 1 and 2). As shown in Table 4, the observed gains suggest that feature fusion strategies are most effective when there is a clear semantic relationship between the source and target datasets. In contrast, performance may be diluted in classes where such relationships are less well defined. The multi-scale feature combination ability of FPN effectively captured these complex lesion patterns. These results indicate that the feature fusion strategy is particularly effective for classes with strong cross-dataset label alignment.

Table 12 and Table 13 summarize the effects of transfer learning between the APTOS and NMC datasets using fine-tuning. For Classes 1, 2 (HE, MA) and Classes 5, 6 (VH, PRH), we present both binary-class scores (individual performance for each lesion type) and multi-label scores (combined performance when these lesion types co-occur). This dual presentation reflects the mapping relationships between APTOS and NMC datasets as defined in Table 4. The model pre-trained on the NMC dataset (nmc-aptos) achieved the most significant performance improvement in APTOS Class 3 (Severe NPDR), increasing from 0.3462 to 0.4483. This improvement is likely due to the semantic alignment between Severe NPDR and the complex co-occurrence of retinal hemorrhages (NMC Class 1) and microaneurysms (NMC Class 2) in the NMC dataset. Conversely, when the model was pre-trained on APTOS dataset (aptos-nmc), substantial performance improvements were observed, particularly in Classes 5 and 6, with multi-label performance improving from 0.2000 to 0.7077. This result can be interpreted from two perspectives. First, APTOS’s PDR (Class 4) directly maps to vitreous hemorrhages (NMC Class 5) and preretinal hemorrhages (NMC Class 6), enabling severity-based learning to contribute to the identification of severe lesions effectively. Second, the pre-trained information from APTOS compensated for the data scarcity in Classes 5 and 6, which occupy a relatively small proportion of the NMC dataset. Additionally, the performance improvement in the combined HE and MA classes (multi-label performance improving from 0.8518 to 0.8629) suggests that the severity classification framework in APTOS also supports recognizing the combined patterns of retinal hemorrhages and microaneurysms.

These findings confirm that cross-dataset transfer is most effective when there is a clear and clinically coherent correspondence between severity levels and lesion types. The severity-based APTOS and the lesion-centered NMC datasets can be used complementarily to address data scarcity and enhance the recognition of complex lesion patterns. However, the effectiveness of this approach depends heavily on the clarity of the mapping relationships between the datasets, as well as the distribution and complexity of classes. Therefore, the choice of transfer strategy should be guided by dataset-specific characteristics, including label alignment, class imbalance, and lesion diversity.

### 6.6. Visualization for Model Focusing

We employed two widely used explainability techniques—GradCAM [46] and IG [47]—to interpret model decision-making and assess its sensitivity to clinically meaningful lesion features. These methods were applied to models trained independently on the APTOS and NMC datasets, as well as to the FT-APTOS model, which was pretrained on NMC and fine-tuned on APTOS. To highlight the most salient activation patterns, only the top 30% of each model’s response map was visualized.

For objective comparison, expert ophthalmologists manually annotated the APTOS fundus images at the pixel level. Each lesion type was marked using distinct colors: hard exudates (black), hemorrhages (red), microaneurysms (green), and cotton wool spots (yellow). These annotations provided reference boundaries to evaluate whether the models’ attention aligned with known diagnostic markers.

**Figure 5:**
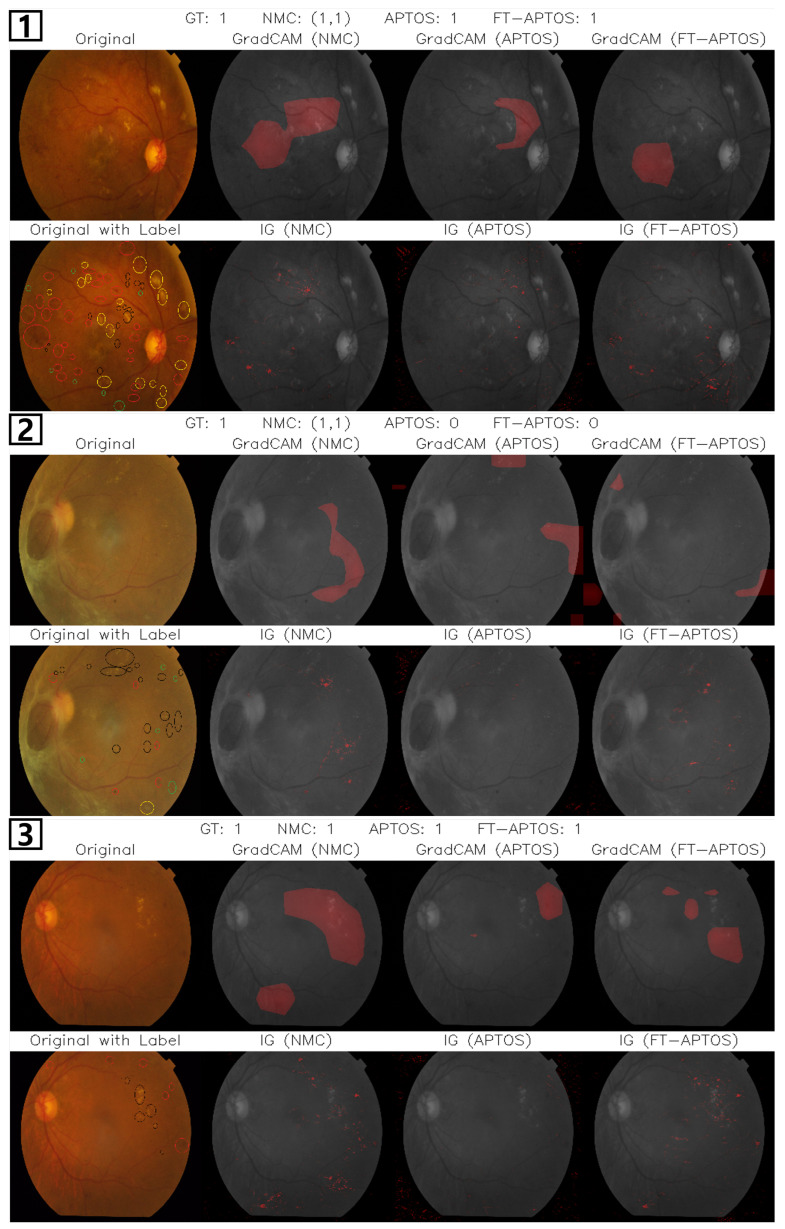
Interpretability comparison on APTOS images (images **1**–**2**: Class 3 Severe NPDR, image **3**: Class 2 Moderate NPDR) using GradCAM and Integrated Gradients (IG) for models trained on APTOS, NMC, and the FT-APTOS model pretrained on NMC and fine-tuned on APTOS. Visualizations highlight the top 30% of activation maps. Pathological features in retinal images are color-coded: hemorrhages (red), microaneurysms (green), hard exudates (black), and cotton wool spots (yellow).).

Figure 5 presents visualization results comparing models trained on APTOS, NMC, and a model pretrained on NMC and fine-tuned on APTOS (FT-APTOS) across different DR severity classes. In the first example of Class 3 (Severe NPDR), which was correctly classified by all models, the NMC-trained model precisely highlighted hemorrhages and microaneurysms—both key diagnostic markers of Severe NPDR—in the IG maps. In contrast, the APTOS-trained model showed broader attention dispersed across background regions, indicating weaker localization of relevant lesions. The FT-APTOS model exhibited sharper focus in IG visualizations, aligning more closely with annotated lesion clusters. These results suggest that lesion-centered pretraining enhances spatial selectivity toward clinically meaningful features, thereby improving severity classification accuracy.

In the second case of Class 3, only the NMC-trained model made an accurate prediction, clearly reflected in the visualizations. Visualizations from the APTOS-trained model exhibited weak and spatially diffuse activation across background regions, failing to capture key pathological features. The FT-APTOS model showed improved attention to detail in lesion location in the IG results. However, the GradCAM visualizations revealed weak activation in these regions.

In our third example featuring Class 2 (Moderate NPDR), all three models correctly classified the image. However, the Integrated Gradients visualizations reveal a significant improvement in attention focusing with the FT-APTOS model. While the NMC-trained model attends to non-diagnostic lesions and the APTOS-trained model focuses primarily on several lesions, the FT-APTOS model demonstrates an enhanced ability to prioritize clinically significant regions while suppressing attention to less important areas. This suggests that the combined knowledge from both datasets enables the model to more effectively distinguish between diagnostically relevant and irrelevant features, resulting in more precise focus on the hemorrhagic lesions that define Moderate NPDR.

Overall, IG results show that the NMC-trained model focuses sharply on lesions like hemorrhages and microaneurysms, closely matching expert annotations. The APTOS-trained model displays broader, less specific attention as it concentrates primarily on severity criteria rather than precise lesion identification, resulting in insufficient feature focusing for its classification decisions. When both approaches are combined in the FT-APTOS model, we observe more refined localization, with the lesion-specific knowledge from NMC complementing the severity-based approach of APTOS to support more informed model decision-making. This suggests that lesion-focused pretraining improves clinical feature alignment and classification accuracy by enhancing the model’s ability to identify the specific pathological features that drive severity assessment. While these findings demonstrate the benefits of combining approaches, challenges remain in fully bridging lesion detection and severity classification, suggesting opportunities for future research using mechanisms like multi-task learning or hierarchical supervision.

## 7. Discussions

Our analysis of DR diagnosis dataset utilization strategies revealed significant insights into the complementary nature of lesion-centered and severity-based approaches. EfficientNet demonstrated 0.138 higher performance than Swin Transformer, particularly in severe case identification, validating CNNs architectures’ advantages in capturing DR’s local characteristics. This hierarchical feature extraction effectively captured multi-scale features from microaneurysms to vascular pattern changes, mirroring clinical examination processes. Classification strategy experiments showed that optimal approaches vary based on lesion pattern complexity and data availability. For Severe NPDR (Class 3) with complex lesion patterns, binary classification yielded a 0.067 improvement despite limited data, suggesting independent learning better captures simultaneous presentations of various lesions. Conversely, PDR (Class 4) showed 0.053 higher performance with multi-class approaches, indicating contextual learning benefits well-defined pathological features. For the NMC dataset, label-chain learning improved performance in data-scarce Classes 1 and 5 by 0.027 and 0.048, respectively, while decreasing in data-rich Classes 2 and 3, confirming that relationship-based learning benefits classes with limited data. The complementarity between lesion-centered (NMC) and severity-based (APTOS) approaches was demonstrated through knowledge transfer experiments, with performance improvements of 0.078 in mild cases with BiFPN [63] and 0.185 in severe cases with FPN [62]. This mirrors clinical practice where detailed lesion identification informs overall severity grading. However, visualization analysis revealed a critical insight: while NMC pre-training enhanced detailed lesion recognition (shown by IG [47]), these features were not always effectively utilized in classification (indicated by GradCAM [46]), suggesting a persistent gap between lesion identification and comprehensive severity assessment.

### 7.1. Clinical Implications

Our findings suggest that AI systems for DR diagnosis should incorporate both lesion-centered and severity-based information to maximize clinical utility. The significant performance improvements in severe case identification could enhance triage of patients requiring urgent intervention, potentially improving clinical outcomes. The visualization analysis showed that models pre-trained on lesion-centered data develop enhanced attention to clinically relevant details, supporting more explainable AI diagnoses that could increase clinician trust.

### 7.2. Limitations and Future Directions

A significant limitation is the restricted accessibility of the NMC dataset due to privacy regulations. While we acknowledge the limitation of single-expert annotation in the NMC dataset, the privacy constraints necessitate practical alternatives to mitigate potential annotation bias. Our approach of combining NMC with the publicly available, multi-validated APTOS dataset provides a pragmatic solution, where the multi-expert validated knowledge from APTOS offers complementary verification for NMC annotations through transfer learning frameworks. Alternatives include synthetic data generation, transfer learning frameworks, and federated learning approaches.

The current NMC dataset only indicates lesion presence without capturing quantitative characteristics (count, size) or spatial distribution patterns essential for clinical diagnosis. This impacts its utility for comprehensive DR assessment and integration with severity-based classifications. Clinical criteria like the 4-2-1 rule rely heavily on these spatial aspects, which the current binary presence/absence labeling cannot adequately represent. Future research should focus on:

1. Enhanced Lesion Representation: Developing standardized annotation protocols capturing lesion counts per quadrant, size categorization, density measurements, and proximity relationships between different lesion types.
2. Clinical Criteria Integration: Creating model architectures with components designed to evaluate clinical rules, implementing multi-stage classification systems mirroring clinical diagnostic processes, and integrating deep learning with rule-based systems encoding clinical knowledge.
3. Multi-institutional Validation: Developing comprehensive validation frameworks across diverse patient populations and clinical settings, investigating domain adaptation techniques, and implementing consensus labeling to establish reliable ground truth.

By addressing these directions, we can develop more accurate, clinically relevant, and generalizable AI systems for DR diagnosis that leverage the complementary strengths of lesion-centered and severity-based approaches, ultimately improving patient care and outcomes.

## 8. Conclusions

In this study, we constructed the NMC dataset through medical professionals’ direct lesion interpretation and labeling. We comprehensively examined DR diagnostic strategies utilizing this dataset across four key research questions: model selection, classification strategy, inter-dataset knowledge transfer, and visualization analysis. Our key findings demonstrate that EfficientNet outperformed Swin Transformer, with notable improvements of 24.5% in Proliferative DR classification. For challenging cases with complex lesion patterns, binary classification yielded a 24.0% improvement in Severe NPDR detection, while feature fusion with FPN achieved remarkable 66.3% performance enhancement in severe case classification through cross-dataset knowledge transfer.

Notably, we demonstrated that the NMC dataset’s lesion-centered approach, which independently labels major DR lesions such as retinal hemorrhages, microaneurysms, and exudates, effectively learns key features for DR diagnosis. This complementary approach addresses limitations of existing severity-based datasets, as validated through both performance metrics and visualization analyses. While Integrated Gradients results showed accurate detection of detailed lesions, GradCAM analysis revealed challenges in effectively utilizing these features for comprehensive severity assessment. These improvements in diagnostic accuracy have significant clinical implications, potentially enabling earlier intervention for high-risk patients and reducing preventable vision loss.

Future research will focus on developing mapping systems that consider quantitative and spatial characteristics of lesions, investigating model architectures that integrate local features with global contextual information, and developing learning methodologies that can directly incorporate clinical diagnostic criteria such as the 4-2-1 rule. Additionally, we plan to enhance the NMC dataset to secure richer lesion information and explore methods for effective integration with other datasets, including potential real-time diagnostic applications. While the NMC dataset is not publicly released due to privacy concerns, future work includes exploring frameworks for controlled data sharing or collaborative access under institutional agreements.

## Figures and Tables

**Table 1:** Characteristics of Diabetic Retinopathy Stages and APTOS Labels.

No	Stage	Sub-Stage	APTOS Class	Description
0	None		0	No DR
1–3	NPDR	Mild	1	Microaneurysms only
		Moderate	2	4 or more blot hemorrhages in one hemi-field only
		Severe	3	Any of the following (4-2-1 rule) and no signs of proliferative retinopathy: · Severe intraretinal hemorrhages and microaneurysms in each of four quadrants · Definite venous beading in two or more quadrants · Moderate IRMA in one or more quadrants
4	PDR		4	One or both of the following: · Neovascularization · Vitreous/preretinal hemorrhage

**Table 2:** Class characteristics of the NMC dataset.

No	Class	Description
0	No abnormality	Normal state with no lesions
1	Retinal hemorrhages	Hemorrhages within the retina caused by the rupture of capillaries or blood vessels
2	Microaneurysms	Formation of microaneurysms due to localized expansion of capillaries
3	Exudates	Yellowish-white deposits on the retina caused by the deposition of proteins and lipids
4	Cotton wool spots	White, fluffy lesions resulting from ischemia in the retinal nerve fiber layer
5	Vitreous hemorrhages	Hemorrhages within the vitreous cavity
6	Preretinal hemorrhages	Hemorrhages occurring in front of the retina
8	Laser scars	Scars left behind after laser photocoagulation therapy
9	Media opacity	Visual obstruction caused by media opacity
10	Artifacts	Artifacts or noise generated during the imaging process

**Table 3:** Comparison of APTOS and NMC Datasets.

Aspect	APTOS Dataset	NMC Dataset
Labeling Method	Single-label based on severity (0 to 4, No DR to Proliferative DR)	Multi-label based on multiple lesions
Detailed Lesion Info	No lesion-centered information. Classified solely by severity (e.g., Mild, Moderate).	Includes lesion-centered labels: retinal hemorrhages (1), microaneurysms (2), exudates (3), cotton wool spots (4), vitreous hemorrhages (5), etc.
Data Distribution	- Higher proportion of ‘Mild DR’. - Lacks sufficient severe DR data.	- Ensures diversity in lesion types. - Higher proportion of ‘Severe DR’.
Special Conditions	No labels for special conditions.	Includes labels for special conditions such as Laser scars (8), Media opacity (9), and Artifacts (10).
Clinical Applicability	Suitable for severity prediction but lacks detailed lesion or clinical context representation.	Allows analysis of lesion count and distribution in addition to severity prediction due to detailed lesion information.

**Table 4:** Experimentally defined mapping between APTOS severity classes and NMC lesion classes for cross-dataset knowledge transfer.

APTOS Class	NMC Class Mapping
0 (No DR)	NMC 0
1 (Mild NPDR)	NMC 2 (Only)
2 (Moderate NPDR)	NMC 1 (≥4 in one hemi-field)
3 (Severe NPDR)	NMC 1, NMC 2 (in all four quadrants)
4 (PDR)	NMC 5 or NMC 6

**Table 5:** Training Results for APTOS Dataset (Micro F1-score, Precision, Recall).

Class	Label	Scratch			NMC→APTOS			F1 Diff
		F1	Precision	Recall	F1	Precision	Recall	
0	No DR	0.9832	0.9925	0.9742	**0.9851**	0.9962	0.9742	+0.002
1	Mild DR	0.5155	0.6098	0.4464	**0.5862**	0.5667	0.6071	+0.071
2	Moderate DR	**0.7943**	0.6950	0.9267	0.7516	0.7035	0.8067	−0.043
3	Severe DR	0.2791	0.4286	0.2069	**0.3774**	0.4167	0.3448	+0.098
4	Proliferative DR	**0.7013**	0.8182	0.6136	0.7000	0.7778	0.6364	−0.001
Overall		**0.8339**	–	–	0.8248	–	–	−0.009

The best results are highlighted in bold.

**Table 6:** Training Results for NMC Dataset (Micro F1-score, Precision, Recall).

Class	Label	Scratch			APTOS→NMC			F1 Diff
		F1	Precision	Recall	F1	Precision	Recall	
0	NA	**0.8984**	0.9129	0.8843	0.8946	0.9102	0.8795	−0.004
1	HE	**0.8358**	0.8485	0.8235	0.8289	0.8583	0.8015	−0.007
2	MA	0.8671	0.8726	0.8616	**0.8795**	0.8754	0.8836	+0.012
3	EX	**0.8563**	0.8726	0.8405	0.8451	0.8289	0.8620	−0.011
4	CWS	**0.6102**	0.7500	0.5143	0.5357	0.7143	0.4286	−0.075
5	VH	0.6667	0.8333	0.5556	**0.7143**	1.0000	0.5556	+0.048
6	PRH	**0.5000**	1.0000	0.3333	**0.5000**	1.0000	0.3333	0.000
Overall		0.8321	–	–	**0.8338**	–	–	+0.002

The best results are highlighted in bold.

**Table 7:** Model Performance Comparison on APTOS Dataset (Micro F1-score, Precision, Recall) EfficientNet: EfficientNet_v2_m, SwinTF: Swin_s.

Class	Label	EfficientNet			SwinTF			F1 Diff
		F1	Precision	Recall	F1	Precision	Recall	
0	No DR	**0.9832**	0.9925	0.9742	0.9779	0.9851	0.9708	−0.005
1	Mild DR	0.5155	0.6098	0.4464	**0.5441**	0.3913	0.8036	+0.029
2	Moderate DR	**0.7943**	0.6950	0.9267	0.7726	0.6526	0.9267	−0.022
3	Severe DR	0.2791	0.4286	0.2069	**0.3093**	0.3393	0.2828	+0.030
4	Proliferative DR	**0.7013**	0.8182	0.6136	0.5631	0.6000	0.5315	−0.138
Overall		**0.8339**	–	–	0.8109	–	–	−0.023

The best results are highlighted in bold.

**Table 8:** Model Performance Comparison on NMC Dataset (Micro F1-score, Precision, Recall) EfficientNet: EfficientNet_v2_m, SwinTF: Swin_s.

Class	Label	EfficientNet			SwinTF			F1 Diff
		F1	Precision	Recall	F1	Precision	Recall	
0	NA	**0.8984**	0.9129	0.8843	0.8951	0.9399	0.8542	−0.003
1	HE	0.8358	0.8485	0.8235	**0.8429**	0.8852	0.8030	+0.007
2	MA	**0.8671**	0.8726	0.8616	0.8603	0.8540	0.8667	−0.007
3	EX	0.8563	0.8726	0.8405	**0.8601**	0.8527	0.8677	+0.004
4	CWS	0.6102	0.7500	0.5143	**0.6202**	0.7917	0.5123	+0.010
5	VH	**0.6667**	0.8333	0.5556	0.5714	1.0000	0.4000	−0.095
6	PRH	**0.5000**	1.0000	0.3333	**0.5000**	1.0000	0.3333	0.000
Overall		**0.8321**	–	–	0.8304	–	–	−0.002

The best results are highlighted in bold.

**Table 9:** Comparison of Classification Strategies on APTOS Dataset (Micro F1-score, Precision, Recall).

Class	Label	Multi-Class			Binary-Class			F1 Diff
		F1	Precision	Recall	F1	Precision	Recall	
0	No DR	**0.9832**	0.9925	0.9742	**0.9832**	0.9962	0.9705	0.000
1	Mild DR	0.5155	0.6098	0.4464	**0.5487**	0.5439	0.5536	+0.033
2	Moderate DR	**0.7943**	0.6950	0.9267	0.7853	0.6814	0.9267	−0.009
3	Severe DR	0.2791	0.4286	0.2069	**0.3462**	0.3913	0.3103	+0.067
4	Proliferative DR	**0.7013**	0.8182	0.6136	0.6486	0.8000	0.5455	−0.053
Overall		**0.8339**	–	–	–	–	–	–

The best results are highlighted in bold.

**Table 10:** Comparison of Classification Strategies on NMC Dataset (Micro F1-score, Precision, Recall).

Class	Label	Multi-Label			Label Chain			F1 Diff
		F1	Precision	Recall	F1	Precision	Recall	
0	NA	**0.8984**	0.9129	0.8843	0.8981	0.9395	0.8602	−0.000
1	HE	0.8358	0.8485	0.8235	**0.8623**	0.8421	0.8834	+0.027
2	MA	0.8671	0.8726	0.8616	**0.8765**	0.8410	0.9151	+0.009
3	EX	**0.8563**	0.8726	0.8405	0.8275	0.8352	0.8199	−0.029
4	CWS	0.6102	0.7500	0.5143	**0.6179**	0.7170	0.5429	+0.008
5	VH	0.6667	0.8333	0.5556	**0.7143**	1.0000	0.5556	+0.048
6	PRH	**0.5000**	1.0000	0.3333	**0.5000**	1.0000	0.3333	0.000
Overall		**0.8321**	–	–	–	–	–	–

The best results are highlighted in bold.

**Table 11:** Knowledge Transfer Effects on APTOS Dataset Using Feature Fusion Methods (Micro F1-score, Precision, Recall).

Class	APTOS Label	APTOS			NMC + APTOS with FPN			NMC + APTOS with BiFPN		
		F1	Precision	Recall	F1	Precision	Recall	F1	Precision	Recall
Class 0	No DR	**0.9832**	0.9925	0.9742	0.9724	0.9706	0.9742	0.9778	0.9814	0.9742
Class 1	Mild DR	0.5155	0.6098	0.4464	0.5490	0.4330	0.7500	**0.5937**	0.5278	0.6786
Class 2	Moderate DR	**0.7943**	0.6950	0.9267	0.7631	0.7086	0.8267	0.7586	0.7160	0.8067
Class 3	Severe DR	0.2791	0.4286	0.2069	**0.4643**	0.4815	0.4483	0.3793	0.3793	0.3793
Class 4	Proliferative DR	**0.7013**	0.8182	0.6136	0.6316	0.5882	0.6818	0.6429	0.6750	0.6136
Overall	–	**0.8339**	–	–	0.8136	–	–	0.8139	–	–

The best results are highlighted in bold.

**Table 12:** Transfer Learning Performance on APTOS Dataset via Fine-tuning (Micro F1-score, Precision, Recall).

APTOS Class	Label	APTOS (Binary-Class)			NMC→APTOS (Binary-Class)		
		F1	Precision	Recall	F1	Precision	Recall
Class 0	No DR	0.9832	0.9962	0.9705	**0.9851**	0.9962	0.9742
Class 1	Mild DR	**0.5487**	0.5439	0.5536	0.5417	0.4432	0.6964
Class 2	Moderate DR	**0.7853**	0.6814	0.9267	0.7600	0.6650	0.8867
Class 3	Severe DR	0.3462	0.3913	0.3103	**0.4483**	0.4483	0.4483
Class 4	Proliferative DR	**0.6486**	0.8000	0.5455	0.6000	0.6667	0.5455

The best results are highlighted in bold.

**Table 13:** Transfer Learning Performance on NMC Dataset via Fine-tuning (Micro F1-score, Precision, Recall).

NMC Class	Label	NMC				APTOS→NMC			
		Binary-Class			Multi-Label	Binary-Class			Multi-Label
		F1	Precision	Recall	F1	F1	Precision	Recall	F1
Class 0	NA	**0.8921**	0.8878	0.8964	-	0.8847	0.8644	0.9060	-
Class 2	MA	**0.8644**	0.8671	0.8616	-	0.8624	0.8393	0.8868	-
Class 1	HE	0.8535	0.8399	0.8676	-	**0.8669**	0.8486	0.8860	-
Class 1, 2	HE, MA	0.8491 0.8545	0.8379 0.8613	0.8609 0.8477	0.8518	**0.8539** **0.8719**	0.8744 0.8832	0.8340 0.8608	**0.8629**
Class 5, 6	VH, PRH	0.2000 0.0000	0.5000 0.000	0.1250 0.000	0.2000	**0.6154** **0.8000**	0.7533 1.0000	0.5217 0.6667	**0.7077**

The best results are highlighted in bold.

## Data Availability

The datasets presented in this article are not readily available because they are part of an ongoing study and contain sensitive patient information. Requests to access the datasets should be directed to haoji@nmc.or.kr.
